# Real time *in vitro *studies of doxorubicin release from PHEMA nanoparticles

**DOI:** 10.1186/1477-3155-7-5

**Published:** 2009-10-20

**Authors:** Raje Chouhan, AK Bajpai

**Affiliations:** 1 Bose Memorial Research Laboratory, Department of Chemistry, Government Autonomous Science College, Jabalpur (MP)-482001, India

## Abstract

**Background:**

Many anticancer agents have poor water solubility and therefore the development of novel delivery systems for such molecules has received significant attention. Nanocarriers show great potential in delivering therapeutic agents into the targeted organs or cells and have recently emerged as a promising approach to cancer treatments. The aim of this study was to prepare and use poly-2-hydroxyethyl methacrylate (PHEMA) nanoparticles for the controlled release of the anticancer drug doxorubicin.

**Results:**

PHEMA nanoparticles have been synthesized and characterized using FTIR and scanning electron microscopy (SEM), particle size analysis and surface charge measurements. We also studied the effects of various parameters such as percent loading of drugs, chemical architecture of the nanocarriers, pH, temperature and nature of the release media on the release profiles of the drug. The chemical stability of doxorubicin in PBS was assessed at a range of pH.

**Conclusion:**

Suspension polymerization of 2-hydroxyethyl methacrylate (HEMA) results in the formation of swellable nanoparticles of defined composition. PHEMA nanoparticles can potentially be used for the controlled release of the anticancer drug doxorubicin.

## Background

The number of reported cases of cancer is steadily increasing in both industrialised and developing countries. The latest world cancer statistics indicate that the number of new cancer cases will increase to more than 15 million in 2020 whereas another report issued by the World Health organization says that there are over 10 million new cases of cancer each year and over 6 million deaths annually are caused by the disease [1]. In spite of the fact that significant progress has been achieved in tumor biology, molecular genetics and in the prevention, detection and treatment of cancer over the last few years, adequate therapy remains elusive due to late diagnosis, inadequate strategies for addressing aggressive metastasis, and the lack of clinical procedures overcoming multidrug resistant (MDR) cancer [2]. The integration of nanotechnology and medicine has the potential to uncover the structure and function of biosystems at the nanoscale level. Nanobiotechnology may provides a reliable and effective tool to treat diseases at a molecular scale. Nanobiotechnology offers an unprecedented opportunity to rationalize delivery of drugs and genes to solid tumours following systemic administration [3]. Examples of nanotechnologies applied in pharmaceutical product development include polymer-based nanoparticles, lipid-based nanoparticles (liposomes, nanoemulsions, and solid-lipid nanoparticles), self-assembling nanostructures such as micelles and dendrimers-based nanostructures among others. In recent years, much research has gone into the characterisation of nanoparticles and their biological effects and potential applications. These include bottom-up and molecular self-assembly, biological effects of naked nanoparticles and nano-safety, drug encapsulation and nanotherapeutics, and novel nanoparticles for use in microscopy, imaging and diagnostics [4].

To be successful a cancer treatment approach needs to overcome physiological barriers such as vascular endothelial pores, heterogeneous blood supply, heterogeneous architecture to name just a few [5], and and it strongly depends on the method of delivery. In the past, many anticancer drugs had only limited success and had major adverse side effects [6,7]. Nanoparticles have attracted considerable attention worldwide because of their unique functional characters such as small particle size, high stability, lower toxicity, tuneable hydrophilic-hydrophobic balance and the ability to bear surface features for target specific localization, etc. Thus, polymeric nanoparticles constitute a versatile drug delivery system [8], which can potentially overcome physiological barriers, and carry the drug to specific cells or intracellular compartments by passive or ligand-mediated targeting approaches [9]. The use of some polymers also allows, at least in principle, to achieve controlled release and the sustained drug levels for longer periods of time. Numerous biodegradable polymeric nanoparticles made of natural polymers such as proteins or polysaccharides have been tried for drug delivery and controlled drug release. More recently the focus of such studies moved onto synthetic polymers, and much progress have been achieved in this area. Recent examples include, for example polycationic nanoparticles for encapsulation and controlled release of amphotericin B by *Vieria *and *Carmona-Ribeiro *[10]; or encapsulation of curcumin for human cancer therapy by *Maitra et al *[11].

**Figure 1:**
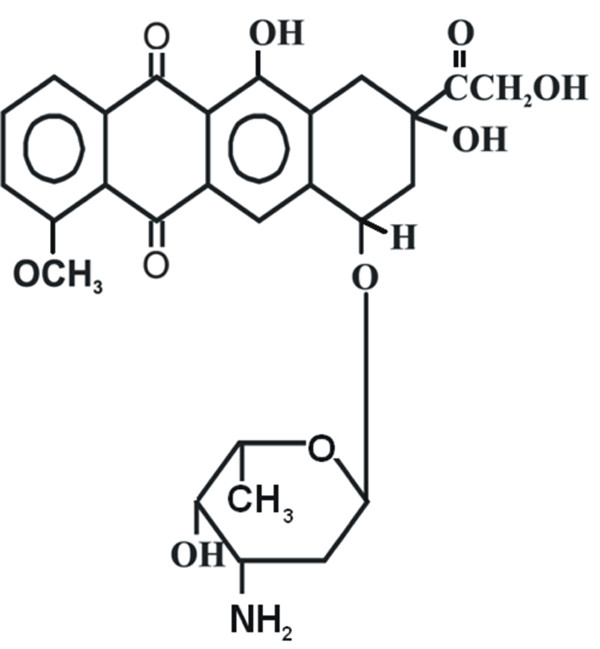
**Structure of drug Doxorubicin**. Chemical structure of anticancer drug [Doxorubicin].

Ideally, a successful nanoparticulate system should have a high drug loading capacity thereby reducing the quantity of matrix material for administration. The drug may be bound to the nanoparticles either (i) by polymerization in the presence of drug in most cases in the form of solution (incorporation method) or (ii) by absorbing/adsorbing the drug after the formation of nanoparticles by incubating them in the drug solution. In the present work we set to further investigate the latter method by studying swelling and controlled release of antitumour drug doxorubicin from synthetic PHEMA nanoparticles. PHEMA attracted significant attention and is well documented in the literature. Many useful properties which make PHEMA attractive for a wide range of biomedical applications [12] include high water content, low toxicity and tissue compatibility. PHEMA has been used in applications such as soft contact lenses [13], drug delivery systems [14], kidney dialysis membranes [15], artificial liver support systems [16] and nerve guidance channels [17]. The presence of polar groups of hydroxyl and carboxyl on each repeat unit makes this polymer compatible with water and the hydrophobic α-methyl groups of the backbone convey hydrolytic stability to the polymer and enhance mechanical strength of the polymer matrix [18]. The drug chosen for this study was doxorubicin hydrochloride, which belongs to the family of anti-tumour drugs (Fig. 1). Doxorubicin is a cytotoxic anthracycline antibiotic [19], it is widely used in the treatment of non-Hodkin's lymphoma, acute lymphoblastic leukemia, breast carcinomas and several other types of cancer. We aimed to design a better PHEMA nanoparticulate delivery system for clinical administration of doxorubicin to achieve higher therapeutic efficacy and reduce side effects, with the overall aim to develop effective oral chemotherapy system.

## Results and Discussion

### Preparation and characterization of Nanoparticles

**Figure 2:**
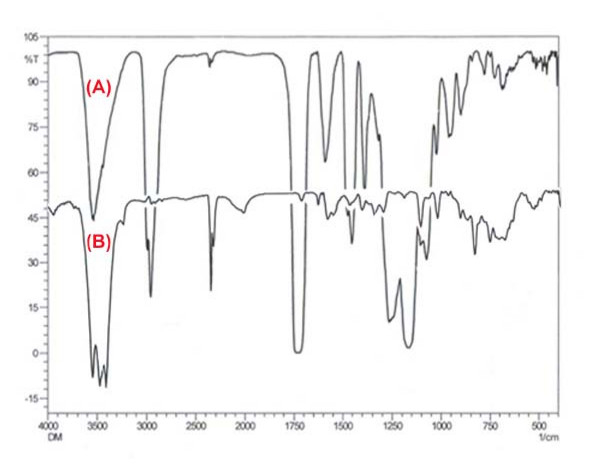
**FTIR spectra of nanoparticles**. FTIR spectra of (a) Pure drug Doxorubicin and (b) Drug loaded PHEMA nanoparticles.

The FTIR spectra of the pure drug (doxorubicin) and loaded nanoparticles are shown in Fig. 2(a) and 2(b), respectively. The IR spectra (b) of loaded nanoparticles clearly indicate the presence of HEMA as evident from the observed bands at 1728 cm^-1 ^(C=O stretching), 1172 cm^-1 ^(O-C-C stretching), 3556 cm^-1 ^(O-H stretching), 2951 cm^-1 ^(asymmetric stretching of methylene group) and 1454 cm^-1 ^(O-H bending) respectively. The spectra (b) also mark the presence of drug (doxorubicin) as evident from the observed bands at 1000-1260 cm^-1 ^(C-O stretching of alcohol) and 675-900 cm^-1 ^(out of plane O-H bending). The resemblance of spectra shown in Fig. 2(a) (the pure drug) and in Fig. 2(b) (loaded nanoparticles) confirms the presence of drug in the loaded nanoparticles.

**Figure 3:**
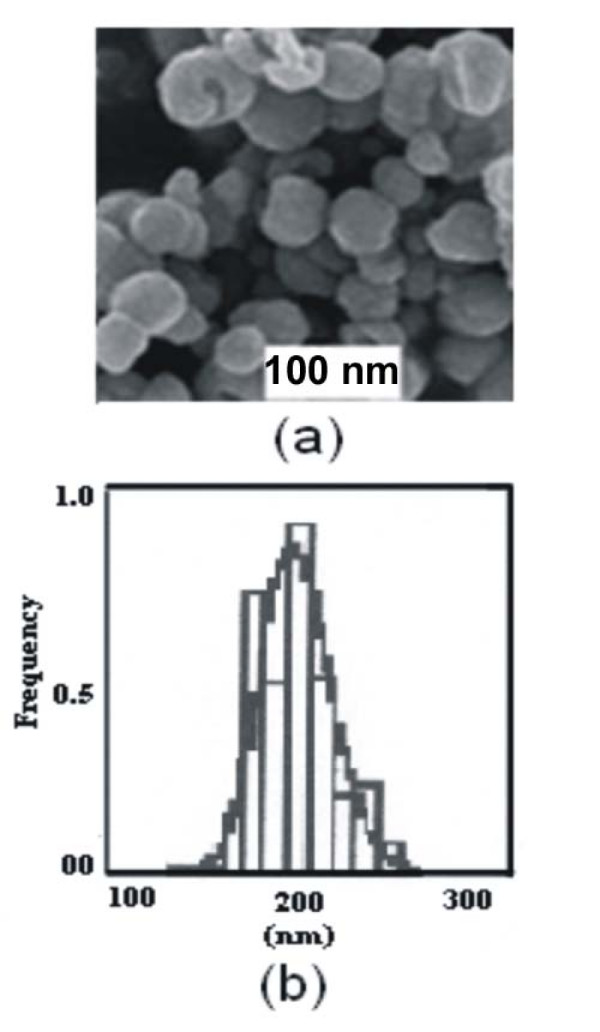
**SEM image and Particle Size distribution curve of nanoparticles**. (a) The SEM images of cross-linked PHEMA nanoparticles and (b) Particle size distribution curve of cross-linked PHEMA nanoparticles.

The SEM image of nanoparticles is shown in Fig. 3(a), which also reveals the morphology of PHEMA nanoparticles. The size of nanoparticles was estimated using SEM images. Under our experimental conditions it has been shown to vary between 100 and 300 nm. The particle size distribution curve of prepared nanoparticles is shown in Fig. 3(b). The small (defined) size of the nanocarriers results in the increased surface to volume ratio [20], enhanced frictional forces as well as adsorption [21]. These properties allow nanoparticles to be held in suspension and largely define their biological fate, toxicity and targeting ability, as well as drug loading potential, their stability and drug release properties. The interaction of nanoparticles with living systems depends on their characteristic dimensions. Previously published studies proved the ability of ultra small nanoparticles to translocate throughout the body (see [22] and references therein).

ξ-Potential is the difference in the electrical charge developed between the dense layers of ions surrounding the particles and the charge of the bulk of the suspended fluid surrounding the particle; it gives information about the overall surface charge of the particles [23]. Thus, the measurements of ξ-potential may indicate the colloidal stability of nanoparticles. The interactions between the particles play an important role in determining colloidal stability. The use of ξ-potential measurements to predict stability attempts to quantify such interactions. Most nanoparticles have a tendency to aggregate which may lead to precipitation that could prove dangerous if those particles are injected intravenously. Since most aqueous colloidal systems are stabilized by electrostatic repulsion, the larger the repulsive forces between particles, the less likely they will come closer together and form an aggregate [24]. Therefore, it is important to know the surface charge, which directly controls aggregation behaviour of the particles in the blood. The values of ξ-potential for unloaded and drug loaded nanoparticles are summarized in Table 1 which clearly indicates that loading of doxorubicin onto nanoparticles surfaces increases positive potential of the nanoparticles surface. The observed increase may be explained by the fact that drug molecules bear a positive charge and due to their loading onto the particle surface the positive charge increases on the surface, which also indicates towards the drug-surface interaction.

**Table 1:** Surface potential of nanoparticles

***pH***	***EMF(mV)***	***EMF(mV)***	***EMF(mV)***
	***Buffer solution***	***Unloaded***	***Loaded***
1.2	276.5	260	280.1
7.4	-34.2	-34.4	-19.9
8.6	-82.8	-86.1	-45.2

Surface potential of unloaded and loaded PHEMA nanoparticles.

*EMF data have been expressed as Mean ± S.D. of at least three determinations.

### Modeling of the release mechanism

The drug loaded PHEMA nanoparticles may be visualized as a three dimensional network of PHEMA macromolecules containing doxorubicin molecules, occupying the free space available between the network chains. During the drug release process, the drug diffuses through the hydrated polymer matrix into the aqueous phase. The process of hydration relaxes the polymer chains and enhances the diffusion of drug molecules. The rate of water uptake (hydration) of polymer particles increases with the hydrophilicity of polymer [25]. The doxorubicin molecules dissolve into water and release out through water permeation channels present in the macromolecular network. The diffusion of doxorubicin molecules and relaxation of PHEMA chains determine the type of release mechanism being followed by the drug molecules. According to Higuchi equation [26] when ***n ***= 0.43, the release is said to be diffusion controlled (Fickian), and when ***n ***= 0.84, the release is said to be non-Fickian (or case II). For ***n ***being in between 0.43 and 0.84, the mechanism becomes anomalous. In some cases ***n ***has been found to exceed 0.84 and the mechanism is known as super case II. The values of D and ***n ***are summarized in Table 2. The data demonstrate that the value of ***n ***is lies between 0.43 to 0.84 in the majority of cases and, therefore, the release of doxorubicin may be considered as non-Fickian and swelling controlled.

**Table 2:** Release exponent and diffusion coefficient of nanoparticles

***S.No***	***HEMA*** ***(mM)***	***EGDMA*** ***(mM)***	***BPO*** ***(mM)***	***pH***	n *	*Dx10*^15^* *cm*^2^ ***min*^-1^**
1	12.37	1.06	0.248	7.4	0.46 ± 0.014	1.81 ± 0.054
2	16.49	1.06	0.248	7.4	0.61 ± 0.018	1.98 ± 0.059
3	20.61	1.06	0.248	7.4	0.50 ± 0.015	1.81 ± 0.054
4	24.73	1.06	0.248	7.4	0.55 ± 0.016	1.69 ± 0.051
5	12.37	0.53	0.248	7.4	0.40 ± 0.012	2.16 ± 0.065
6	12.37	1.59	0.248	7.4	0.44 ± 0.013	2.04 ± 0.061
7	12.37	2.12	0.248	7.4	0.45 ± 0.013	2.04 ± 0.061
8	12.37	1.06	0.082	7.4	0.58 ± 0.017	1.81 ± 0.054
9	12.37	1.06	0.165	7.4	0.47 ± 0.014	2.48 ± 0.074
10	12.37	1.06	0.33	7.4	0.58 ± 0.017	1.98 ± 0.059
11	12.37	1.06	0.248	1.8	0.86 ± 0.026	2.61 ± 0.078
12	12.37	1.06	0.248	8.6	0.60 ± 0.018	1.81 ± 0.054

Data showing the release exponent and diffusion coefficient under varying experimental conditions

*Data have been expressed as Mean ± S.D. of at least three determinations.

### Effect of % loading on drug release

**Figure 4:**
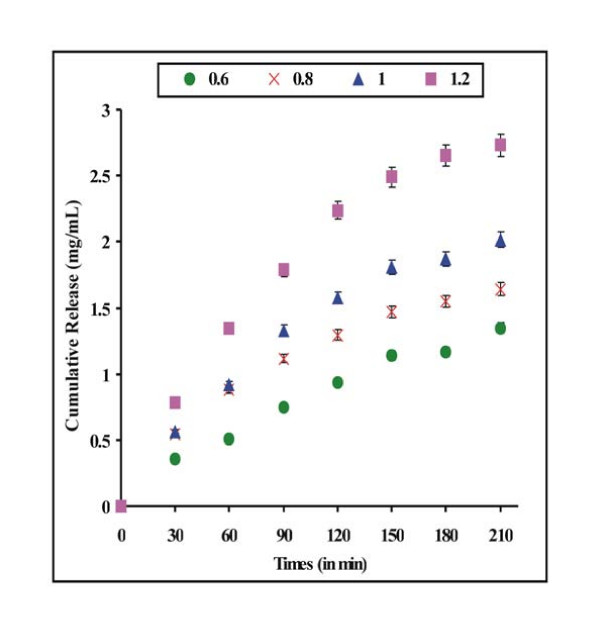
**Effect of % loading of doxorubicin**. Effect of % loading of doxorubicin on its release profiles from loaded nanoparticles of definite composition [HEMA] = 12.37 mM, [EGDMA] = 1.06 mM, [Bz_2_O_2_] = 0.248 mM, pH = 7.4, temp. = 37°C.

An important aspect in using nanoparticles as drug vehicle is the effect of the drug loading levels on the drug release rates. Higher drug loading may be achieved either by using highly concentrated drug solution or repeated soaking of nanoparticles in the drug solution and then drying them. In the present work, nanoparticles of defined composition were loaded with different amounts of doxorubicin by allowing the particles to swell in the drug solution of varying concentrations ranging between 1.2 and 2.4 mg/mL. The loaded particles were then allowed to release the entrapped drug into the release medium. Drug release results are shown in Fig. 4. The amount of released doxorubicin increases with increasing percent loading. Similar results were reported previously by us and others for different drug release systems [27].

### Effect of monomer on drug release

**Figure 5:**
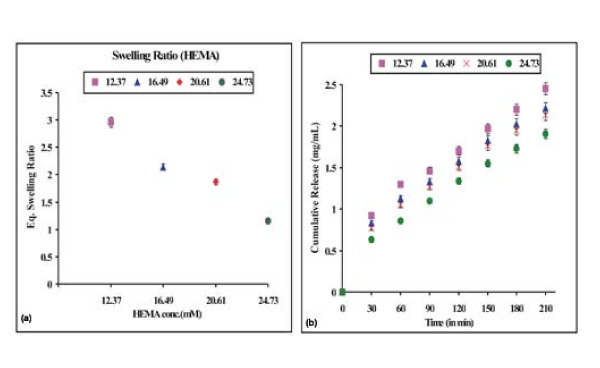
**Effect of monomer [HEMA]**. Effect of monomer [HEMA] content on the (a) swelling profile and (b) release profile of the nanoparticles of definite composition [EGDMA] = 1.06 mM, [Bz_2_O_2_] = 0.248 mM, % loading = 28%, pH = 7.4, temp. = 37°C.

Doxorubicin release profiles are sensitive to chemical architecture of the carrier as well as the experimental conditions used to prepare the drug carrier. The effect of HEMA on the release of doxorubicin has been investigated by varying the monomer concentration in the range 12.3 mM to 24.7 mM. The swelling ratio and release results are shown in Fig. 5(a) and 5(b). Our data indicate that the swelling ratio and cumulative release of doxorubicin decreases with increasing concentration of HEMA. The results may be explained by the fact that as the content of PHEMA increases in the nanoparticles, the polymeric nanoparticles becomes largely crowded with polymer chains and this consequently reduces the free volume accessible to the penetrant water molecules. This obviously brings about a fall in the swelling ratio as well as the released amounts of drug. Another possible reason may be that with increase in PHEMA content the interaction between the polymer chains and the drug molecules increases which also results in a lower release of doxorubicin.

### Effect of Cross-Linker on Drug (Doxorubicin) Release

**Figure 6:**
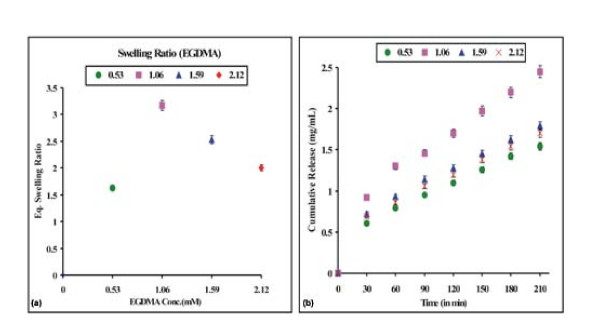
**Effect of cross-linker [EGDMA]**. Effect of cross-linker [EGDMA] content on the (a) swelling profile and (b) release profile of the nanoparticles of definite composition, [HEMA] = 12.37 mM, [Bz_2_O_2_] = 0.248 mM, % loading = 28%, pH = 7.4, temp. = 37°C.

In the cross-linked polymeric structures the swelling process may be controlled by the introduction of an appropriate amount of a second monomer with hydrophobic character. Chemically cross-linked hydrogels have been developed as carrier for drugs [28] in the last decade. Cross-linkers have pronounced effect on the swelling ratio as well as on kinetics of the drug release. We decided to use EGDMA, which is a known hydrophobic cross-linker, as a cross-linking agent in the present study. The effect of the degree of cross-linking on the swelling and drug release has been investigated by varying the concentration of EGDMA in the range of 0.53 to 2.12 mM in the feed mixture of the polymerization recipe. The swelling and release results are shown in Fig. 6(a) and 6(b) respectively. Initially the swelling ratio and drug release increase (up to 1.06 mM of EGDMA). Beyond this concentration, both the swelling ratio and drug release decrease. The observed increase is unusual and may be explained by loosening of the macromolecular chains of the nanoparticles. The latter may be due to the hydrophobic nature of EGDMA and the hydrophobic interactions occuring along EGDMA segments. The observed decrease in the released amounts of drug above 1.06 mM EGDMA could be because of the reduced free volume accessible to water molecules in more densely cross-linked polymers. Similar results have been reported for chitosan hydrogels (by *Singh et al *[29]). We showed earlier that the introduction of a cross-linker increases the glass transition temperature (Tg) of the polymer, which restrains the mobility of network chains at experimental temperature and, therefore, lowers both the amount of water sorption as well as drug release [30].

### Effect of initiator on drug release

**Figure 7:**
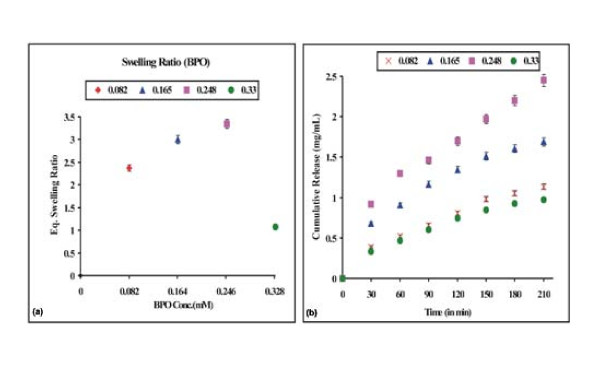
**Effect of initiator [Bz_2_O_2_]**. Effect of initiator [Bz_2_O_2_] content on the (a) swelling profile and (b) release profile of the nanoparticles of definite composition, [HEMA] = 12.37 mM, [EGDMA] = 1.06 mM, % loading = 28%, pH = 7.4, temp. = 37°C.

In free radical polymerization the concentration of initiator has a direct impact on the molecular weight of the polymer [31]. We used Bz_2_O_2_ as a polymerization initiator and its concentration in the reaction mixture was varied in the range of 0.082 - 0.330 mM. Swelling and release results are depicted in Fig. 7(a) and 7(b). An initial increase in concentration of Bz_2_O_2_ in the range of 0.082 - 0.248 mM results in an increased swelling as well as drug release. The increase in the concentration of initiator may bring about an increase in the number of primary free radicals, which may eventually result in lower molecular weight of the PHEMA. Since a polymer with lower molecular weight has lower hydrodynamic volume in aqueous solution, the PHEMA chains acquire greater mobility and, therefore, show increased swelling and increased drug release. However, the higher concentration of the initiator results in shorter PHEMA chains and smaller mesh size of the polymer network and reduced drug loading and release.

### Effect of pH on drug release

Since the pH change occurs at many specific or physiological sites in the body, it is one of the important parameters in the design of drug delivery systems. Several methods have been proposed for targeting the specific regions. Among these, utilization of pH changes within the GI tract and exploitation of bacterial enzymes localized within the colon are of especial interest for the controlled drug delivery [31]. Differences in pH in the target site may allow a specific drug to be delivered to that target site only. The underlying principle for targeted drug delivery is the pH controlled swelling of hydrogel which normally results from the change in relaxation rate of network chains with changing pH of the medium. The pH profile of normal tissue is different from that of pathological tissues such as cancerous and infected tissues. *Amiji et al *[32] reported that pH of normal tissue is higher than the pH of infected and tumourous tissues. The physical properties of stimuli-responsive carriers such as swelling/deswelling, particles disruption and aggregation vary according to changing environmental conditions. These change the nanocarriers-cells interactions, and therefore the release of the drug at tumour site may be achieved. Drug loaded nanoparticles undergo rapid dissolution and release the drug content in the acidic microenvironments of a tumour [33].

**Figure 8:**
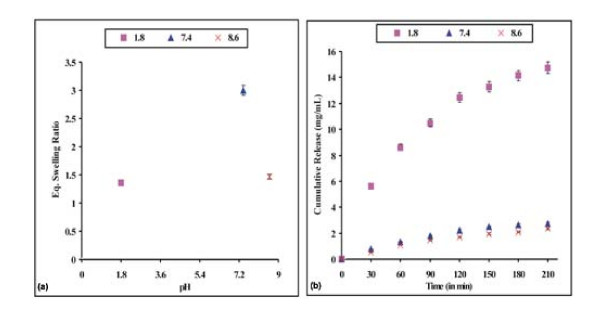
**Effect of pH**. Effect of pH on the (a) swelling profile and (b) release profile of the nanoparticles of definite composition [HEMA] = 12.37 mM, [EGDMA] = 1.06 mM, [Bz_2_O_2_] = 0.248 mM, % loading = 23%, temp. = 37°C.

In the present work, the release dynamics of the doxorubicin have been studied under varying pH conditions. The results are shown in Fig. 8(a) and 8(b). We found that less drug is released at physiological and alkaline pH, whilst the most efficient release is achieved at acidic (pH = 1.2) conditions. These results are not fully consistent with the swelling results. The unloaded PHEMA nanoparticles show maximum swelling at physiological pH solution, whilst loaded PHEMA nanoparticles show maximum release at acidic pH. The reason behind the lower swelling of unloaded nanoparticles in acidic and alkaline conditions is that the nanoparticles do not swell sufficiently in acidic and alkaline solutions. Drug loaded nanoparticles swell better in the acidic solution rather than at physiological pH or under alkaline conditions. Similar results have been reported recently for pH-sensitive liposomes [34]. These were stable at physiological pH of 7.4, but degraded to release active drug in target tissues in which the pH is less than physiologic values, such as in the acidic environment of tumour cells.

### Effect of temperature on drug release

**Figure 9:**
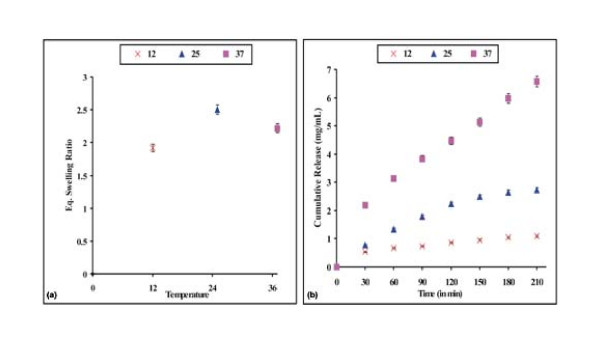
**Effect of temperature**. Effect of temperature of the release medium on the (a) swelling profile and (b) release profile of the nanoparticles of definite composition [HEMA = 12.37 mM, [EGDMA] = 1.06 mM, [Bz_2_O_2_] = 0.248 mM, % loading = 28%, pH = 7.4.

Temperature sensitivity is one of the most important characteristics in drug delivery technology. It has a direct influence on the swelling and release behaviour of a hydrogel. Temperature affects both the segmental mobility of the hydrogel chains as well as the diffusion of penetrant molecules. In this study, the effect of temperature on the swelling ratio and drug release through PHEMA nanoparticles has been investigated by varying the temperature of the swelling medium in the range of 12 - 37°C. The results are summarised in Fig. 9(a) and 9(b). Swelling increased with temperature up to 25°C, but decreased above this temperature. However, cumulative release was highest at 37°C. The increased temperature results in faster relaxations times of the polymer network due to the increased kinetic energy, which facilitates water sorption process [35].

### Effect of physiological fluids on drug release

**Figure 10:**
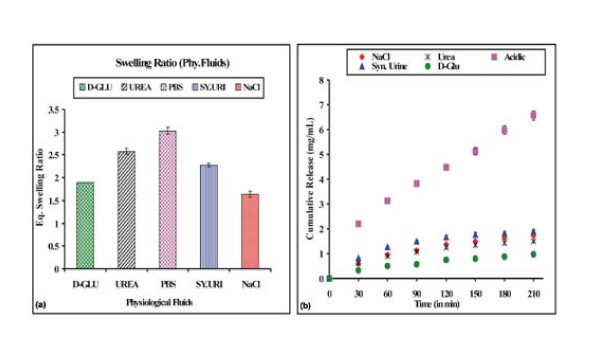
**Effect of physiological fluids**. Effect of physiological fluids on the (a) swelling profile and (b) release profile of the nanoparticles of definite composition [HEMA] = 12.37 mM, [EGDMA] = 1.06 mM, [Bz_2_O_2_] = 0.248 mM, % loading = 28%, pH = 7.4, temp. = 37°C.

The influence of solutes on the swelling behaviour and doxorubicin release kinetics was examined by performing swelling and release experiments in the presence of solutes such as urea (15% w/v) and D-glucose (5% w/v) and in physiological fluids such as saline water (0.9% NaCl) and synthetic urine. The results are shown in Fig. 10(a) and 10(b). The presence of additives reduces both the swelling ratio as well as drug release. Salts present in the release medium are likely to reduce the osmotic pressure in the system thus resulting in lower extent of swelling of loaded nanoparticles and the drug release.

### Chemical stability of the entrapped doxorubicin

**Figure 11:**
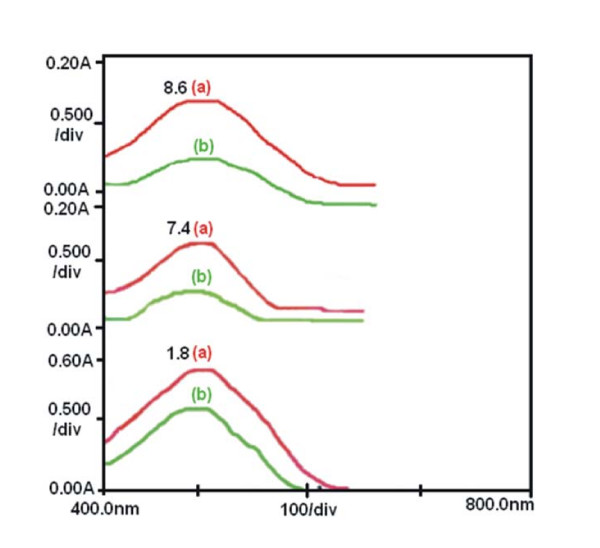
**Chemical stability of Doxorubicin**. UV spectra showing the chemical stability of doxorubicin in its pure solution (a) and released media (b) at different pH (1.8, 7.4, 8.6).

The chemical stability of the entrapped drug was investigated by recording the UV-visible absorbance spectra of pure doxorubicin and the drug released into the release medium at different pH (fig. 11). There are no noticeable differences in the obtained absorbance spectra at all pH tested (pH1.8, pH7.4, pH8.6), suggesting no significant changes in the physical properties of the drug, and most likely of its chemical structure during nanoparticle loading and drug release.

## Conclusion

The PHEMA nanoparticles can be prepared by suspension polymerization method and characterized by techniques such as FTIR, SEM and particle size analysis. The addition of model drug, doxorubicin, to polymeric nanoparticles results in 28% drug entrapment. Release profiles of doxorubicin can be greatly modified by varying the experimental parameters such as percent loading of doxorubicin and concentrations of HEMA, cross-linker and initiator. Swelling of nanoparticles and the release of doxorubicin increases with the increase in percentage loading of drug. The amount of released drug decreases with increasing HEMA and EGDMA content of the nanoparticles. Increase in the concentration of the initiator, benzoyl peroxide, from 0.082 mM to 0.248 mM results in the increase of drug release, but this effect is reduced at higher concetration of benzoyl peroxide. The best combination of individual components for making PHEMA nanoparticles for doxorubicin delivery was 12.37 mM HEMA, 1.06 mM EGDMA and 0.248 mM Bz_2_O_2_. Fast drug release was observed at acidic pH1.2 at 37°C whilst physiological and alkaline pH and lower temperature slow down the release of doxorubicin. alts and additives affecting osmotic pressure also suppress the extent of drug release. Absorption spectra of doxorubicin do not change following its capture and release form the nanoparticles, indicating that chemical structure of the drug is likely to be unaffected by the procedure.

## Methods

### Materials

2-Hydroxyethyl methacrylate (HEMA) and ethyleneglycol dimethacrylate (EGDMA) were purchased from Sigma Aldrich Co. USA. Benzoyl peroxide (BPO) (MERCK) and polyvinyl alcohol (PVA) (Mol. Wt. 14000) (MERCK) were used as the initiator and the stabilizer, respectively. Toluene (MERCK) was used as the diluent. All chemicals were of analytical grade and doubly distilled water was used throughout the experiments.

### Methods

HEMA monomer was purified by using a previously reported method [30]. The purity of distilled HEMA was determined by high-pressure liquid chromatography (HPLC), [Backmen System (Gold 127)] equipped with a ultraviolet detector and a 25 cm × 46 mm id separation columns ODS (C_18_) of 5 μm particle size. The UV detector was set at 217 nm. The mobile phase was methanol-water (60:40 v/v) and the flow-rate was kept at 1 mL/min. All samples were diluted with pure methanol to 1/1600. 10 μL samples were injected for each analysis. Samples of known concentrations of MAA and EGDMA were injected into the HPLC and the resultant chromatogram was used to construct a standard curve of known concentrations vs. area under the curve. The chromatogram showed two distinct peaks. The first peak, at 3.614 min was identified as MAA. The next peak at 5.503 min was the major peak due to HEMA monomer. The amounts of impurities of MAA and EGDMA in the monomer samples were found to be less than 0.01 mol% MAA and 0.001 mol% EGDMA.

Preparative methods for making nanoparticles for pharmaceutical use are broadly divided into two categories, those based on physiochemical properties such as phase separation and solvent evaporation [36], and chemical reactions such as polymerization [37], and polycondensation. In the present study cross-linked PHEMA nanoparticles of defined composition were prepared by using a modified suspension polymerization technique, as previously reported by *Kaparissides et. al*. [38]. In particular, the polymerization was carried out in an aqueous phase containing PVA, which was used as the stabilizing agent. The mixture containing the 12.37 mM HEMA (the monomer), 1.06 mM EGDMA (the cross-linker) and 0.248 mM Bz_2_O_2 _(the initiator) dispersed in toluene was added into 500 mL conical flask containing the suspension medium (200 mL aqueous PVA solution (0.5% W/V)). The reaction mixture was flushed by bubbling nitrogen and then sealed. The reaction mixture was then placed on magnetic stirrer and heated by vigorous stirring (600-700 rpm) at 80°C for 2 h and then at 90°C for 1 h. The cross-linking reaction was completed within three hours. After cooling, the polymeric particles were separated from the polymerization medium by washing thrice with toluene and twice with acetone. The collected nanoparticles were dried at room temperature to obtain the fine white powder and thereafter stored in airtight polyethylene bags.

The IR spectra of cross-linked PHEMA nanoparticles were recorded on a FTIR spectrophotometer (Perkin-Elmer, 1000 Paragon) (Shimadzu). While recording FTIR spectra KBr disc method was used for preparation of samples.

Morphological studies of cross-linked PHEMA nanoparticles were performed on scanning electron micrographs (SEM). SEM observations were carried out with a Philips, 515, fine coater. Drops of the polymeric nanoparticles suspension were placed on a graphite surface and freeze dried. The sample was then coated with gold by ion sputter. The coating was performed at 20 mA for 4 min, and observation was made at 10 KV. Nanoparticles were further characterized by particle size analysis for size and size distribution. The particle size analysis of prepared nanoparticles was performed on a particle size analyzer (Malvern Mastersizer 2000).

Zeta potential studies were performed with a digital potentiometer (Model No. 118, EI Product, Mumbai, India). In a typical experiment 0.1 g nanoparticles were dispersed in 20 mL of respective pH solution and emf was recorded using a compound electrode system. A similar experiment was also repeated for drug-loaded nanoparticles.

Swelling properties of hydrogels can be used as a method to trigger drug release [39]. Swelling of nanoparticles was studied by a conventional gravimetric procedure. In a typical experiment, 0.1 g of nanoparticles were allowed to swell in a definite volume (10 mL) of PBS taken in a pre weighed sintered glass crucible (pore size 5-10 μm) and weighed after a definite period by removing excess PBS by vacuum filtration. The swelling process of nanoparticles was monitored continuously up to 15 min after which no weight gain of swollen nanoparticles was recorded which clearly indicates equilibrium swelling condition. The weight swelling ratio of nanoparticles was calculated from the following equation,

(1)

where, W_t _is the weight of swollen nanoparticles at time t, and W_0 _is the initial weight of dry nanoparticles (at time 0).

For loading of drugs onto nanoparticles, a known volume of drug doxorubicin was taken and diluted with the appropriate amount of PBS solution and shaken vigorously for mixing of drug and PBS solution. Drug-loaded nanoparticles were prepared by swelling 0.1 g of nanoparticles in freshly prepared drug solution (10 mL) until equilibrium swelling was reached. The % loading of drug onto nanoparticles was calculated by the following equation:

(2)

where, W_d _and W_0 _are the weights of loaded and unloaded nanoparticles, respectively.

*In-vitro *release of the loaded doxorubicin was carried out by placing the dried and loaded nanoparticles (0.1 g) in a test tube containing a definite volume (10 mL) of phosphate buffer saline (PBS) as the release medium (pH = 7.4) (1.2 mM KH_2_PO_4_, 1.15 mM Na_2_HPO_4_, 2.7 mM KCl, 1.38 mM NaCl). The amount of doxorubicin released from the polymeric nanoparticles was measured spectrophotometrically at 496 nm (Shimandzu 1700 Phama Spec.) and the released amount of drug was determined from the calibration plot.

To study the kinetics of the release process, drug-loaded nanoparticles were added to the release medium and the suspension was shaken for 3.5 h. For monitoring the progress of the release process, aliquots were withdrawn at desired time intervals and the amount of drug released was estimated spectrophotometrically.

The drug release from polymeric nanoparticulate systems is actually the combination of Fickian (diffusion) and non-Fickian movements [40] of drug molecules through polymer chains. In the present study the kinetic data were analyzed with the help of the following equation, which could be helpful in determining the mechanism of the release process,

(3)

where W_t _and W_∞ _are the amount of the drug release at time t and at infinity time (equilibrium amount of drug released), respectively, and K is rate constant. The constants K and n are characteristics of the drug-polymer system. An introduction to the use and the limitations of these equations was fist given by Peppas et al. [41]. For evaluating the diffusion constant of loaded drugs, the following equation can be used:

(4)

where, D is the diffusion constant of the drug and L being the diameter of the dry nanoparticles.

In order to check the chemical stability of entrapped drug in different release media, the UV spectral studies (Shimandzu 1700 Pharma Spec) were performed as described in [42].

All experiments were done at least thrice and a fair reproducibility was observed. The data summarized in Tables have been expressed as mean ± SD of at least three independent determinations. The plots were drawn taking the mean values and each curve has been shown to include error bars.

## Competing interests

The authors declare that they have no competing interests.

## Authors' contributions

RC synthesized PHEMA nanoparticles and performed in vitro functional assays, AKB conceived the idea of nanoparticles, guided to conduct the studies, supervised data analysis and authored the manuscript.

## References

[B1] Stewart BW, Weihues P (2003). World Cancer Report: World Health Organizaton.

[B2] Sinha R, Kim GJ, Nie S, Shin DM (2006). Nanotechnology in cancer therapeutics: bioconjugated nanoparticles for drug delivery. Mol Cancer Ther.

[B3] Nie S, Xing Y, Kim GJ, Simons JW (2007). Nanotechnology applications in cancer. Annu Rev Biomed Eng.

[B4] Soloviev M (2007). Nanobiotechnology today: focus on nanoparticles. J Nanobiotechnol.

[B5] Jain RK (2001). Delivery of molecular and cellular medicine to solid tumours. Adv Drug del Rev.

[B6] Priya Pathak, Katiyar VK (2007). Multi-Functional Nanoparticles and Their Role in Cancer Drug Delivery - A Review. Azonano.

[B7] Cho K, Wang X, Nie S, Chen ZG, Shin DM (2008). Therapeutic Nanoparticles for Drug Delivery in Cancer. Clin Cancer Res.

[B8] Frank G, Langer R, Farokhzad OC (2008). Precise engineering of targeted nanoparticles by using self-assembled biointegrated block copolymers. PNAS.

[B9] Vashir JK, Reddy MK, Labhasetwar VV (2005). Nanosystems in Drug Targeting: Opportunities and Challenges. Current Nanosci.

[B10] Vieira DB, Carmona-Ribeiro AM (2008). Cationic nanoparticles for delivery of amphotericin B: preparation, characterization and activity *in vitro*. Journal of Nanobiotechnology.

[B11] Maitra A (2007). Polymeric nanoparticle-encapsulated curcumin ("nanocurcumin"): a novel strategy for human cancer therapy. Journal of Nanobiotechnology.

[B12] Michalek J, Pradny M, Arthyukhov A, Sloufm M (2005). Macroporous hydrogels based on 2-hydroxyethyl methacrylate Part III Hydrogels as carriers for immobilization of proteins. J Mater Sci Med.

[B13] Manneti C, Casciani L, Pescosolido N (2002). Diffusive contribution to permeation of hydrogel contact lenses: theoretical model and experimental evaluation by nuclear magnetic resonance techniques. Polymer.

[B14] Ruiz J, Mantecon A, Cadiz V (2002). Investigation of loading and release in PVA-based hydrogels. J Appl Polym Sci.

[B15] Ostrovidova GU, Makeev AV, Shamtsiam MM (2003). Polyfunctional film coatings for medical use. Mater Sci Eng C.

[B16] Mao J, Zhao L, Deyao K, Shang Q, Yang G, Cao Y (2003). Study of novel chitosan-gelatin artificial skin *in vitro*. J Biomed Mater Res.

[B17] Dalton PD, Flynn L, Schoichet MS (2002). Fibre templating of poly (2-hydroxyethyl methacrylate) for neural tissue engineering. Biomaterials.

[B18] Chou KF, Han CC, Lee S (2000). Water transport in crosslinked 2-hydroxyethyl methacrylate. Polym Eng Sci.

[B19] Reddy LH, Murthy RSR (2004). Pharmacokinetics and biodistribution studies of doxorubicin loaded poly (butyl cyanoacrylate) nanoparticles synthesized by two different techniques. Biomed Papers.

[B20] Hede S, Huilgol N (2006). Nano: the new nemesis of cancer. J Cancer Res Ther.

[B21] Moghimi SM, Hunter AC, Murray JC (2005). Nanomedicine: current status and future prospects. FASEB J.

[B22] Hoet PHM, Brüske-Hohlfeld I, Salata OV (2004). Nanoparticles - known and unknown health risks. Journal of Nanobiotechnology.

[B23] Lu W, Zhang Y, Tan Y-Z, Hu K-L, Jiang X-G, Fu S-K (2005). Cationic albumin-conjugated PEGlyted nanoparticles as novel drug carrier for brain delivery. J Cont Rel.

[B24] Jahanshahi M, Babaei Z (2008). Protein nanoparticle: A unique system as drug delivery vehicles. African J Biotech.

[B25] Higuchi T (1961). Rate of release of medicaments from ointments bases containing drugs in suspension. Pharmaceut Sci.

[B26] Budhian A, Seigel SJ, Winey KI (2008). Controlling the in-vitro release profiles for a system of haloperidol-loaded PLGA nanoparticles. Int J Pharm.

[B27] Bajpai AK, Rajpoot M (2000). Release and diffusion of sulphamethoxazole through acrylamide-based hydrogel. J Appl Polym Sci.

[B28] Risbud MV, Hardikar AA, Bhat SV, Bhonde RR (2000). pH-sensitive freeze-dried Chitosan-polyvinyl hydrogels as controlled release system for antibiotic delivery. J Control Release.

[B29] Singh AA, Narvi SS, Dutta PK, Pandey ND (2006). External stimuli response on a novel chitosan hydrogel crosslinked with formaldehyde. Bull Mater Sci.

[B30] Bajpai AK (2004). Adsorption of a blood protein on to hydrophillic sponges based on poly(2-hydroxyethyl methacrylate). J Mater Sci Mater Med.

[B31] Bajpai AK, Mishra A (2005). Preparation and characterization of tetracycline-loaded interpenetrating polymer networks of carboxymethyl cellulose and poly(acrylic acid): water sorption and drug release study. Polym Int.

[B32] Ganta S, Devalpally H, Shahiwala A, Amiji M (2008). A review of stimuli-responsive nanocarriers for drug and gene delivery. J Cont Rel.

[B33] Devalpally H, Shenoy D, Little S, Langer R, Amiji M (2007). Poly (ethylene oxide)-modified poly (epsiloncaprolactone) nanoparticles for targeted delivery of tamoxifen in breast cancer. Cancer Chemother Pharmacol.

[B34] Wang X, Yang L, Chen Z, Shin DM (2008). Application of Nanotechnology in Cancer Therapy and Imaging. CA Cancer J Clin.

[B35] Bajpai AK, Saini R (2005). Preparation and characterization of spongy cryogels of poly(vinyl alcohol)-casein system: water sorption and blood compatibility study. Polym Int.

[B36] El-Shabouri MH (2002). Positively charged nanoparticles for improving the oral bioavailability of cyclosporine. Int J Pharm.

[B37] Zhang Q, Zhen Z, Nagai T (2001). Prolonged hypoglycemic effect of insulin-loaded polybutyl cynoacrylate nanoparticles after pulmonary administration to normal rats. Int J Pharm.

[B38] Kaparissides C, Alexandridou S, Kammona O, Dini E (2002). Polymeric nano- and microparticles for controlled release applications. Workshop of CPERI.

[B39] Peppas NA, Langer R (2004). Origins and development of biomedical engineering within chemical engineering. AICHE Journal.

[B40] Budhian A, Seigel SJ, Winey KI (2005). Production of haloperidol loaded PLGA nanoparticles for extended controlled drug release of haloperidol. J Microencapsul.

[B41] Siepmann J, Peppas NA (2001). Modeling of drug release from delivery systems based on hydroxypropyl methyl cellulose. Adv Drug Deliv Rev.

[B42] Wang LF, Chen WB, Chen YB, Lu SC (2003). Effects of preparation methods of hydroxyl propylmethyl cellulose polyacrylic acid blended films on drug release. J Biomater Sci Polymer Edn.

